# An Evaluation of the Relationship Between Critical Thinking and Creative Thinking: Complementary Metacognitive Processes or Strange Bedfellows?

**DOI:** 10.3390/jintelligence13020023

**Published:** 2025-02-18

**Authors:** Christopher P. Dwyer, Deaglán Campbell, Niall Seery

**Affiliations:** Technology Education Research Group (TERG), Department of Technology Education, Technological University of the Shannon: Midlands Midwest, N37 HD68 Westmeath, Ireland

**Keywords:** critical thinking, creative thinking, problem-solving, reflective judgment, metacognition, heuristics

## Abstract

Though both critical thinking and creative thinking are often cited and promoted as important cognitive processes in personal, academic and professional settings, common misconceptualisation of both often leads to confusion for non-experts; for example, with respect to lumping them together erroneously as much the same thing, completely confusing them for one another or, on the other hand, distinguishing them to such an extent that all genuine overlap is ignored. Given the importance of these processes in real-world scenarios, quality education and ‘metaeducation’ is necessary to ensure their appropriate translation to students and educators, alike. Thus, the aim of the current review is to discuss various perspectives on both critical and creative thinking with particular focus paid to addressing such common misconceptualisation. Detailed discussion is provided with respect to important ways in which they are distinct and overlap. Recommendations for what contexts application of each are appropriate are also provided. Furthermore, implications for such enhanced understanding are discussed, in light of both theory and research.

## 1. Introduction

Both critical thinking and creative thinking are often cited as being efficacious tools for personal, academic and professional performance (e.g., Saiz et al. 2024). With respect to the former, research indicates that critical thinking (hereon stylised as ‘CritT’) facilitates better judgment and decision-making (Gambrill 2006), less dependence on cognitive bias and heuristic thinking (Facione and Facione 2001; McGuinness 2013) and a more complex understanding of target information (Dwyer et al. 2012; Halpern 2014), and enhances the likelihood of achieving better grades, employability and becoming more informed and more active citizens (Barton and McCully 2007; Holmes and Clizbe 1997; National Academy of Sciences et al. 2005). Outcomes facilitated by creative thinking (hereon stylised as ‘CreaT’), cognitive speaking, are those associated with cognitive flexibility, sensitivity to problems, idea fluency and novelty (Guilford 1950; Plucker et al. 2004); and, more broadly, societal, technological and economic innovation (Sawyer 2012; Shalley et al. 2009; Smith-Bingham 2006; Sternberg and Lubart 1999), as well as successful adaptation for daily and/or complex decision-making or problem-solving (e.g., see Karunarathne and Calma 2024; Lindberg et al. 2017; Morris and König 2020; Smith-Bingham 2006; Verzat et al. 2017); with the World Economic Forum calling creative thinking the skill poised for the most rapid growth in significance in the years ahead. Indeed, the World Economic Forum (2023) further identified analytical thinking (i.e., largely commensurate with *critical thinking*) and creative thinking as the top two most important skill sets as rated amongst employers.

Though such recognition of both forms of thought is abundant in research and educational policy, it remains that quality education and ‘meta-education’ (i.e., teacher education, with respect to teaching educators, or future educators, about education; e.g., passive learning through classes on educational theory and domain-specific topics to be taught; and active learning through teacher training) is necessary to ensure the appropriate translation of these metacognitive processes for educators, so that they may, in turn, teach these to their students, regardless of academic domain. Despite recommendations for the enhancement of CritT and CreaT throughout educational and post-educational life, clearer understanding of these two processes, particularly with respect to how they overlap and how they are distinct, remains necessary to facilitate this translation. Thus, the aim of the current review is to evaluate such similarities and differences and to make recommendations for how best to make their relationship clearer in educational settings, so that they may be successfully applied in personal, educational and professional settings.

The impetus for this review is, to a large extent, based on common misconceptualisation of both thinking processes (e.g., see Dwyer 2017); specifically, the trend where many might ‘lump’ CritT and CreaT together into the same family of thought or (meta)cognitive processes because of surface level similarities (see below). While research suggests that there is certainly some overlap between the two processes, it also suggests that they are importantly distinct (Wechsler et al. 2018). Indeed, such common collation practices—where academics and educators draw substantial links between the two (e.g., consider a kind of a ‘critico-creative thinking’^1^; Fisher 2001)—might actually reflect some nuances associated with the special issue on the two^2^ processes in which this manuscript appears.

In other cases, some might *completely* confuse one thought process for the other. Such mistakes are, arguably, frustrating to those whose research focus and mission is founded in one and/or the other; but at the same time, they are ultimately forgivable. Both processes are forms of metacognition that have ‘thinking’ as part of their terminology, immediately preceded by another word that starts with ‘Cr-’; and both forms of thinking are often used with the goal of developing a solution to a problem (indeed, problem-solving is a core application of both). However, these are just surface level features. Thus, when accounting for these similarities in a concise manner, one sees both as forms of thinking, of a metacognitive nature, with the common goal of generating solutions to problems, in which both start with ‘Cr-’, followed by ‘thinking’. Again, confusion, in context, is forgivable. What is not forgivable, however, is for educational programmes and institutions to promote these in a manner that simply pays ‘lip service’ to trends in learning outcomes, without (1) investing resources into either ensuring staff are adequately knowledgeable in these areas to teach them or (2) providing training opportunities for such knowledge gain. Whereas the similarities are perhaps most observable at surface level, as described above, the distinctions become more evident the ‘deeper’ one goes in an effort to better understand the nature of these two thought processes (as would be accomplished through the recommended training).

Notably, such misconceptualisation issues are of particular concern in educational settings where thorough and clear description of what ‘needs to be done’ (with respect to thinking) is necessary for success in those arenas. For example, clear exposition of to what CritT refers is of utmost importance in research methodology and academic writing, where a clear meaning of CreaT is vital in areas pertaining to Engineering and Design (which are, in turn, importantly distinct from perspectives in fine-art or fiction writing)^3^.

## 2. What Are They?

CritT is a metacognitive process, consisting of a number of skills and dispositions, that, through purposeful, self-regulatory reflective judgment, increases the chances of producing a logical solution to a problem or a valid conclusion to an argument (Dwyer 2017, 2020, 2023; Dwyer et al. 2012, 2014, 2015, 2016; Dwyer and Walsh 2019; Quinn et al. 2020). Such a definition has been used in both cognitive psychology and educational settings. On the other hand, CreaT has been described as a metacognitive process, wherein the convergence of intellectual abilities, knowledge, styles of thinking, personality, motivation and the environment may, subsequently, yield a solution or conclusion that is (1) unusual or novel and (2) appropriate or valuable (Halpern 2014; Runco and Jaeger 2012; Sternberg 2003, 2010). Notably, such a description is often used in the cognitive psychology literature. A more, ‘education-focused’ definition might refer to CreaT as an iterative, dynamic process (e.g., involving exploration, refinement and synthesis, underpinned by goal setting, reflection and adaptability), bridging domain-specific expertise and domain-general skills, facilitated by lived experience, CritT and collaboration, which enables navigation of problem-spaces towards realisation of outcomes that are not only original but also appropriate and contextually relevant (e.g., see Baynes 1992, 2009; Lucas 2016; Rhodes 1961; Smith and Smith 2010).

In accounting for definitions of both, it is useful to conceptualise them as distinct. However, understanding of both is necessary if they are to be considered valued learning outcomes. For example, misconceptualisation concerns do not end at the ‘lumping of the processes together’; some might perceive such distinction as extending beyond what may be the case. Indeed, specialists/researchers in CreaT may erroneously confuse CritT for something it is not, as might be the case for CritT experts to confuse CreaT for something it is not. Thus, the following sections present turns taken ‘playing devil’s advocate’ (Dwyer 2023) from the perspective of individuals who may be working from a commonly misconceptualised or incomplete understanding of the processes. Notably, such explication is by no means an attempt to suggest that one form of thinking is superior to another; rather, its purpose is to both: exemplify how one might be led to think in light of misconceptualisation or incomplete understanding; and provide nuanced perspectives on how the two differ. Further worth noting is that such misconceptualisations are not necessarily exclusive to each form of thought and may, indeed, apply to both or even to thinking more broadly. In any case, misconceptualisations are presented contextually as examples where further consideration is required. Following this exercise, discussion will turn to exhibition of how these processes do, indeed, overlap and complement one another in particular contexts, despite their important distinctions.

### 2.1. Misconceptualised Issues with Creative Thinking

#### 2.1.1. Is There a Threshold That Consitutes Creative Thinking?

CritT is engaged when an individual wants to inform their decision-making in relation to topics of concern, in which case, such thinking is applied with caution, in order to ensure logic every step of the way (Dwyer 2017, 2023, forthcoming). Such is a core foundation of CritT—the activation of reasonable and logical thinking. Herein lies the first potentially common misconceptualised difference between the two forms of thought—consider this example of misconceptualisation: *in CreaT, it is not a prerequisite that the thinking must be applied to topics the thinker is personally concerned about, nor does it have to be logical, because the goal is not necessarily trying to establish a rationale that is true and reasonable; for example, CreaT might be applied in a leisurely manner, such as making up an interesting story or answering a fantastical ‘what if…’ type of question.*

This misconception highlights an important consideration: just because thinking utilises some aspect(s) of CreaT, does not mean that it qualifies as CreaT. Similarly, just because some level of ‘criticism’ is applied in what might otherwise be low effort thinking, does not make it critical thinking. Simply, though there is ‘good thinking’ and ‘bad thinking’ (i.e., depending on contextual criteria; e.g., outcomes or process), it would be inaccurate to say ‘good CritT’ or good ‘CreaT’ because each process was either appropriately applied (i.e., not just picking-and-choosing some aspect[s] of the process) or it was not (e.g., see Dwyer 2017, forthcoming). For example, where appropriate application of CreaT might require substantial effort, reflexive responses (Lieberman 2003) like that considered above (see also, System 1 thinking; Stanovich and West 2000) might simply represent some form of ‘auto-heuristic idea-generation’ (consider Bargh 1989; Higgins 1989; Tversky and Kahneman 1983, in context). Thus, even though some level of ‘creation’ might be used in these low effort examples, that does not make the process used CreaT—at least, not from the standpoint of the evidence-based conceptualisation provided above. According to Paul and Elder (2008, pp. 5–7).

“[an idea] that roams aimlessly through half-formed images, that meanders without an organizing goal, is neither creative nor critical…. A mind that does not systematically and effectively embody intellectual criteria and standards is not disciplined in reasoning things through. Such a mind is not creative.”

#### 2.1.2. Feasibility and Appropriateness

Another example where something akin to auto-heuristic idea-generation is distinct from CreaT pertains to the issue of feasibility. For example, if the goal is to be ‘novel or unusual’, it might be reasonable to conclude that feasibility is not necessarily a priority. Though this, of course, is fine when telling a ‘creative’ story full of plot-holes, inconsistency and/or requires substantial suspension of belief, it is problematic in cases of problem-solving where utility of a creative solution would be questionable if it does not conform to practical, moral, political, economic requirements. From a CritT perspective, in order for a solution to be selected for application, it needs to be feasible. But, this is also the case for CreaT, as solutions generated need to be *appropriate*—the goal of CreaT is to arrive at the most appropriate solution for the problem in context. This may mean incremental innovation over radical innovation, where the goal state in seeking a novel solution supersedes any personal feelings/biases etc.—consistent with the education-based perspective above; wherein a contextual value judgement, aligned between the intentional act and goal state, is made that determines the output’s ‘fitness for purpose’.

#### 2.1.3. Experience and Originality

With that, one genuine issue CritT perspectives might find troublesome with commonly considered notions of CreaT is its reliance on experience as a cornerstone for idea generation. The trouble with personal experience, as an information source, is that it is not particularly credible, given that it is often based on a sample size of one. Each and every individual is unique and thus, so too are their experiences. One cannot generalise the experience of one to larger populations. In terms of CritT, sources like personal experience and anecdotal evidence are among the first to be eliminated from further consideration. Research-based evidence is the gold standard for what is to be considered when CritT is engaged (Dwyer 2017). With respect to CreaT, though, experience is paramount to idea generation. It can be argued that CreaT often relies on and celebrates such intuitive judgment for idea generation; that is, the opposite of reflective judgment (and, in turn, the fundaments of CritT) with respect to Cognitive Continuum Theory (e.g., see Cader et al. 2005; Dunwoody et al. 2000; Hamm 1988; Hammond 1981, 1996, 2000). In this context, individual experience might represent ‘fuel’ for creative ideation, which facilitates novel or unusual solutions in the first instance and creates a socio-emotive resonance between people (Sinek 2011). According to Sinek (2011), such intuitive judgments are, perhaps, governed less by explicit evidence-based rationale and more by when things ‘feel’ right. Moreover, people develop experience-based heuristics for everyday problems to simplify the cognitive load and decision fatigue associated with constant thought (e.g., Baumeister 2003; Sweller 2010; Vohs et al. 2014). These experience-based protocols are used to remind the thinker what happened in a similar, past situation and brings to mind what they did to generate a solution in context (Kahneman 2011). Such approaches (independent of both CritT and CreaT) are often useful and work most of the time, but when they fail, they fail ‘big’; and, furthermore, when an evidence-based rationale is required, such heuristics do not suffice.

With that, CritT perspectives are not so inflexible that they fail to recognise that humans learn through experience. Even the gold standard source of information, research-based evidence, must be learned from somewhere, through human experience. However, over-use of experience can potentially hinder CreaT, over time, as a result of unwanted pattern formation, for example, in terms of CreaT’s requirement of *originality* (i.e., not in absolute terms [*can any idea be completely novel?*], rather, an output from a personal creation, an artefact of thought, that has value and originated from a process of synthesis and refinement). Such pattern formation can be problematic with respect to idea generation associated with problem-solving, in which case, one’s ‘freedom to create’ (as often [mis]interpreted as being paramount to CreaT, see below) actually restricts the developer in light of such interests, biases, emotions, etc. This example is reminiscient of Maslow’s hammer (i.e., ‘when all you have is a hammer, it is tempting to treat everything as if it were a nail’) and exhibits people’s tendency to approach CreaT and problem-solving from an implicitly biased perspective (Dwyer, forthcoming). Consistent with use of the often criticised availability heuristic (Kahneman 2011; Tversky and Kahneman 1974), people often rely on the (cognitive) resources available, easily adaptable and accessible to them for use, regardless of the relationship between an actual scenario and how one perceives it to be.

However, context is key (see below for further elaboration). The conceptualisation of originality used here accounts for the fact that little (if anything) can be completely novel, thus accounting for potential pattern formation. The crucial element of this consideration is how does it account for it? The answer to this question, likewise, succeeds in addressing much of the overall concern surrounding experience: such potential criticism is based on an incomplete description; thus, the potential for misconceptualisation. While CreaT *does* engage experience as a potential evidence-based for idea-generation, it *does not* treat it the same as the aforementioned auto-heuristic idea-generation approach. That is, CreaT does not use experience as an evidence-base and then simply apply the solution; rather, it takes information from experience and then assesses the subsequent idea (e.g., through simulated trial-and-error) in an evaluatory manner akin to that of CritT. Just as CritT is a process, so too is CreaT, wherein various ‘checkpoints’ are engaged relative to ensuring the aforementioned ‘fitness of purpose’.

Notably, the issue of domain-specificity also plays an important role in the manner one might think about ‘experience’. For example, consider naturalistic decision-making and the recognition-primed model in terms of how highly-experienced/expert individuals make domain specific, practical decisions in real-world settings (Klein 2008). In light of Klein’s research (e.g., Klein 1989, 2008), the recognition-primed model describes how people apply their experience/expertise in the form of a repertoire of patterns (Klein et al. 1986), which organize the primary variables operating in the situation and allow for quick identification of relevant environmental cues, potential outcomes, possible goals and, subsequently, selection of an appropriate reaction(s). Ultimately, when faced with a problem within or relevant to one’s expertise domain, one can (quickly) apply learned patterns to the current situation; thus, facilitating rapid, experience-based decisions of importance that are accurate (Klein 2008).

#### 2.1.4. Popular ‘Sub-Types’

One final conceptual issue, with respect to ‘critiquing’ CreaT pertains to the various ‘sub-types’ of CreaT that have been popularised over the past 50 or so years. Perhaps, the popularisation of such sub-types (and their potential misinterpretation) is among the core issues with which perspectives, like those from CritT standpoint, take ‘umbrage’. Consider, for example, the notions of *lateral thinking* and *thinking outside-of-the-box*.

In contrast to CritT, lateral thinking is more concerned with the migration of thinking from what is known across the spectrum of possibilities (rather than reflective inference of ‘reasonable’ solutions or conclusions); and engages the provocation and generation of ideas, as well as the selection of an idea as a means of breeding and applying new associated ideas (De Bono 1967, 1985). For example, consider the following scenario and what has transpired:

A man in a pub walks up to the bar and asks the bartender for a pint of water. The bartender pulls a gun on the man, points it at him and cocks it. The man looks at the bartender and says sincerely, ‘Thank you’.

One may develop a number of possible explanations in this lateral thinking puzzle, given that multiple explanations fit (in light of the information provided), with some better than others. Arguably, what might make such an approach attractive to thinkers is that it can be engaged following previously failed attempts at solving problems where ‘digging deeper’ may not work; thus, facilitating change of the approach’s direction (i.e., *laterally*). De Bono describes lateral thinking in terms of comparing real-world problem-solving to a game of chess, where game pieces are not given to us, but instead created.

From a CritT perspective, the problem with this perspective is one of credibility, relevance and/or logical strength. In CritT, if the requisite ‘pieces’ are not available (i.e., information), one cannot just ‘create it’ (i.e., without some justifiable evidence-base). Granted, lateral thinking facilitates the creation of a solution when no other means of thought might be able to provide one. But, where this may seem like a strength on the surface, critical thinkers would much rather report not knowing (e.g., in terms of Socratic ‘knowing’ or the Dunning-Kruger effect (Kruger and Dunning 1999)), indicating that further research is necessary or search for help or additional resources to ‘obtain the necessary pieces’. Simply, they would rather no answer than to provide one that could be incorrect or incomplete.

Furthermore, such puzzles (as that presented above) provide poor examples, at a conceptual level, because they often have only one solution provided (i.e., in this case, the man has hiccups and the bartender attempts to scare them away)—despite multiple solutions that might fit—which questions the true ‘laterality’ of such thinking. With that, De Bono’s approach to higher-order cognition, like lateral thinking, has received a great deal of criticism, particularly as a result of focusing primarily on the creation and development of ideas rather than the reliability, validation and efficacy of the lateral thinking approach (Moseley et al. 2005; Sternberg and Lubart 1999). Moreover, regardless of the potential value of lateral thinking in some contexts, it shares very little in common with CritT (with respect to this discussion of CritT and CreaT overlap) other than that they both require some element of reflection.

With respect to thinking ‘outside-the-box’ (TOTB), it is perhaps the romanticised view of the approach that is problematic, particularly in relation to the implication that one’s thinking is not restricted or bound by some governing force (hence, ‘outside-the-box’)—it implies a freedom of thought and expression of ideas. Though the origin of the phrase or those similar is debated, there is record of similar use dating back to the late 1800s (Annual Register 1888). Though various descriptions of the process exist, the common theme among them that is consistent with the definition of CreaT used here pertains to the generation of an idea that is original, novel and/or unusual. Perhaps, though, that is where much of their similarity ends.

For example, the issue of appropriateness consistent with CreaT is, arguably, not as apparent in common descriptions of TOTB. Moreover, there are other CreaT approaches that are inconsistent with TOTB (e.g., see thinking *inside*-the-box below). Furthermore, the aforementioned notion of freedom is not necessarily a useful characteristic of many forms of CreaT (e.g., in terms of concepts like boundaries).

**Figure 1:**
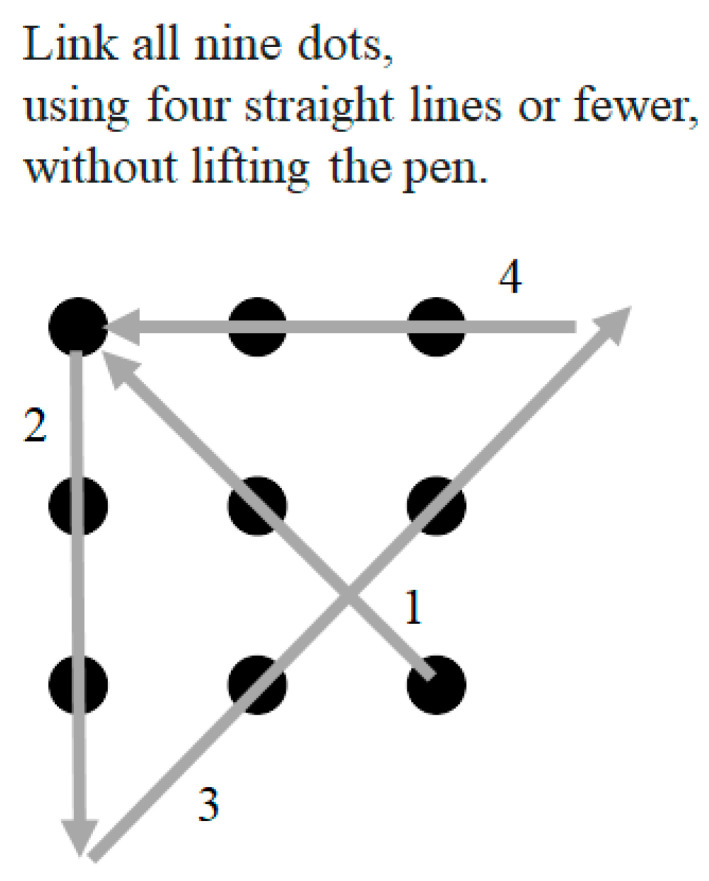
Nine-dot problem with solution.

Indeed, one of the oldest examples of TOTB, still used today, is that of the nine-dot problem (J. F. Anderson 1954; see Figure 1), where the thinker must literally extend their effort beyond the boundary to solve the problem. However, consistent with discussion above, such ‘freedom’ may not always be helpful, especially in problem-solving scenarios. In addition to the notion of such aforementioned ‘freedom’ potentially leading to self-restriction in terms of experience-based bias, it must also be considered that the freedom from ‘parameters’ applied to thinking may not necessarily be a good thing either, particularly in situations that require CritT. Parameters may facilitate process-oriented thinking in a beneficial manner. Consider problem-solving theory with respect to recommended steps one might take so develop a solution to a problem (see also, Dewey 1910; Halpern 2014; Hopwood 1974). Following an organised list of steps is far from ‘free-thinking’; yet, problem-solving is one of the classic examples of opportunities for a TOTB approach to CreaT. Even within the landscape of problem-scenarios, being provided ‘givens’ and ‘options’ provide much needed context for solution-generation.

Moreover, though TOTB can be useful in certain contexts, it remains that there’s nothing ‘wrong’ with thinking *inside*-the-box (e.g., where extant research might represent an established information set from which to extrapolate a foundation for problem-solving, which, arguably, fits the bill quite well of what might substantiate being inside-the-box). Likewise, in application, an important consideration of TOTB should be its frequency of use. It can be argued that seldom, in real-world scenarios, are people ever truly provided opportunities to TOTB. Sure, individuals might be encouraged to do so, but then at the same time, they are often landed with caveats and guidelines (be it explicitly or implicitly). In a practical manner, it is useful to consider such parameters, caveats and guidelines not as restrictions to thought; rather, as *resources*. Indeed, situational constraints may foster creativity in the context of inside-the-box thinking (Le Cunff 2022). Consistent with discussion regarding concepts surrounding Maslow’s hammer, individuals use what they have—what is accessible and available—and what one has can be of great value with respect to idea generation and problems-solving. For example, ‘givens’ and ‘options’ associated with problem scenarios provide thinkers with parameters for working within closed conditions (e.g., Dwyer 2017; Halpern 2014; Hopwood 1974). In such cases, TOTB might wrongfully ignore the one thing that can help the thinker (e.g., dismissing guidelines of project briefs in an effort to TOTB and exhibit one’s ‘CreaT’).

With that, when considering misconceptualisations associated with CreaT, it is important to remember that *context is key*. Just as CritT is recommended for contexts when engagement with an established evidence-base is necessary for important decisions (and perhaps time-consumingly unnecessary in other scenarios), the ‘criticisms’ presented here particularly apply in contexts where CritT might be a better fit than the proposed ‘CreaT’ being used in isolation. Indeed, there are contexts where CreaT is the preferred approach and CritT may be problematic.

### 2.2. Misconceptualised Issues with Critical Thinking

#### 2.2.1. Inflexibility

One potentially common misconceptualisation of CritT, relative to CreaT, regards its flexibility. Indeed, on one end of the spectrum, it is often the case that people overestimate their ability to think critically and the frequency in which they do it (see Dwyer, forthcoming). On the other hand, CritT might be misconceptualised as being inflexible and only useful in certain contexts (i.e., only applicable when there is an actual evidence-base from which to work). However, the reality lies somewhere in between. Dwyer (2017, 2023) indicates that CritT should be applied only in situations where the individual is genuinely concerned about the outcome of their thinking, when there is sufficient time to engage the resources that require it and when one is rested and motivated to engage. However, the very same can be said about CreaT. Indeed, these recommendations apply to any form of cognition where ‘good thinking’ is desired.

Though these features might distinguish CritT from other forms of low effort thinking or auto-heuristic idea-generation, the genuine issue for consideration here is that CreaT might exhibit superior flexibility in situations that require an answer (i.e., where abstaining from decision-making is not an option), where problem-solvers are faced with either a substantially lacking research evidence-base (in which the requisite knowledge may not be readily available for application) or other constraints/insufficiencies to engage with what is there. This is not to say that CritT cannot be applied in such situations (rather, the reflective judgment component of CritT might take the load of decision-making responsibility from that of evaluating extant research), but it does form a scenario in which CreaT can work well to ‘save the day’. That is, given that outcomes generated by CreaT are likewise logical and appropriate, CreaT can act as a reasonable substitute when an evidence-base is either lacking or speculative.

Indeed, this notion harkens back to the long-established adage, *necessity is the mother of invention*. Such a position provides two implications for consideration. First, it implies that, in some contexts, CreaT can be a useful alternative when CritT is not feasible. Second, CreaT can be used alongside the CritT component of reflective judgment (i.e., an individual’s purposeful, self-regulated consideration and understanding of the nature, limits, and certainty of *knowing*; how this can affect how they defend their judgments and reasoning in context; and acknowledgement that their views might be falsified by additional evidence obtained at a later time; Dwyer 2023; Dwyer et al. 2015; King and Kitchener 1994, 2004). With that, such engagement of reflective judgment—and the success of its use alongside CreaT—is largely dependent on how well-developed it is. If it is not ‘well-developed’, then it might be reasonably argued that CreaT offers a solution in situations that might require CritT, but CritT cannot facilitate (i.e., in situations where ‘I do not know’ is an unacceptable outcome, even though such abstinence from decision-making is an acceptable CritT outcome [akin to good epistemological practice; e.g., Socratic Knowing]).

#### 2.2.2. Emotion

Further to CritT not always being feasible to apply, another issue CreaT perspectives might take with CritT is the, arguably, overly idealistic treatment of emotion. From a CritT standpoint, ‘good thinking’ is conducted void of emotion (as much as it can be possible), given that it is an artefact of bias and experience. Indeed, a large body of research indicates a negative impact of emotion on decision-making and higher-order cognition (e.g., see Anticevic et al. 2011; Chuah et al. 2010; Denkova et al. 2010; Dolcos and McCarthy 2006; Kahneman and Frederick 2002; Slovic et al. 2002; Strack et al. 1988). However, it remains that no individual’s thinking can ever be completely void of emotion or associated bias, given the subjective, individualistic nature of a person’s thinking. Thus, where CritT perspectives can recommend avoiding emotion (as much as possible), true success of such recommendation (i.e., total elimination of emotion from thinking) is near impossible. Instead, CreaT perspectives acknowledge the nature of human emotion for what it is, alongside its association with bias and experience, and embraces this (i.e., CreaT is open-minded to emotive rationales just as it is to research-based justification). In some contexts, creative thinkers are encouraged to limit their emotion, experience and bias (though, perhaps not to the extent that CritT might); in others, they are encouraged to engage it. In either case, it can be said that CreaT provides a more ‘realistic’ treatment of emotion and, likewise, one of CreaT’s true ‘selling-points’ is its empathy, flexibility and adaptability, depending on the situation.

## 3. Context Is Key

The notion of context is addressed and discussed throughout this article in two important ways. First, it is discussed with respect to conceptualisation; that is, in terms of all of this ‘depending on’ how one conceptualises both CritT and CreaT, hence the importance of providing detailed descriptions, as done so above. Indeed, it is hoped that these are adequately clear with respect to both overlap and important distinctions, as foundations for the discussion that follows. However, if one was to consider, say, a description from Pablo Picasso that “The chief enemy of creativity is good sense”, the problem is much clearer in that the focus of such description seems to emphasise the distinction. Thus, much of this depends on how one conceptualises CritT and CreaT (e.g., see Dwyer 2017; Dwyer et al. 2016; Lloyd and Bahr 2010).

On the other hand, context can also refer to acknowledging that there is a time and a place for everything, including CritT and CreaT. In situations where CreaT is warranted, CritT may not be and vice versa. Likewise, where CritT is required, sometimes CreaT is warranted as well, be it in a manner described here or perhaps even through TOTB, thinking inside-the-box or maybe even lateral thinking. Sometimes, it is not. Ultimately, the most realistic recommendation, perhaps, is one that accounts for people’s tendency to use what they already have/know (e.g., be it experience [potentially-biased] or expertise-based knowledge (see, again, Klein 2008)), the amount of provisional knowledge and/or established evidence available (e.g., givens), and varying levels of motivation to engage additional information about the issue to make a decision or develop a solution (e.g., disposition; see Dwyer et al. 2014, 2016; Dwyer 2020).

### 3.1. Problem-Solving: A Specific Context Where the Overlap Many Assume Exists

In earlier discussion, particularly in the section dealing with potential misconceptualisation issues with CreaT, it might have been noticed that the context of ‘problem-solving’ repeatedly reared its head with respect to being somewhat of a caveat to criticisms pertaining to CreaT. The reason for this, perhaps, is that *problem-solving* represents the context in which there is arguably the most overlap between CritT and CreaT. With that, problem-solving is an application of both CritT and CreaT, referring to a cognitive process that facilitates the ability to identify both the problem at hand and the desired goal, in light of the problem, through generation and selection of potential solutions among alternatives (Dwyer 2017). Given previous definitions and descriptions of CritT, it is perhaps unsurprising that recommended problem-solving processes (see Table 1; see also, Halpern 2014; Hopwood 1974; Robbins et al. 2004) would utilise CritT skills likes analysis, evaluation and inference. However, given the nature of such problem-solving processes, it is also the case that CreaT requires similar skill application. For example, Steps 1 and 2 in this CreaT problem-solving process include CritT processes akin to analysis; Steps 3, 5 and 6 revolve around evaluation; and Steps 4 and 7 refer to processes associated with inference.

Despite their many distinctions, both forms of thinking, when treated as processes (as in the case of problem-solving), share a substantial amount of overlap, beyond that of just the surface-level similarities addressed early in this manuscript. Perhaps one of the best examples of such overlap stems from an important quote, not by a researcher in either field, but rather Steve Jobs, which happens to represent among the best efforts to describe CreaT, known to us, in this context:

Creativity is just connecting things. When you ask creative people how they did something, they feel a little guilty because they didn’t really do it, they just saw something. It seemed obvious to them after a while.

Jobs’ quote succeeds in making clear an important concept. While he explicitly discusses what CreaT is through example (albeit a simplified version), at the same time he is also describing a simplified version of the CritT skill of inference (i.e., drawing a conclusion in light of the gathering of credible, relevant and logically sound information (Dwyer et al. 2014; Facione 1990); the final in the sequence of analysis, evaluation and inference skills). Indeed, inference is consistent with synthesis, cognitively speaking (Dwyer 2011; Dwyer et al. 2014); and synthesis is akin to the ability to create (L. W. Anderson and Krathwohl 2001; Bloom et al. 1956; Dwyer et al. 2014). That is, we ‘create’ by synthesizing information we have previously analysed and evaluated, so that a logical and feasible conclusion/solution may be inferred (Dwyer 2017).

**Figure 2:**
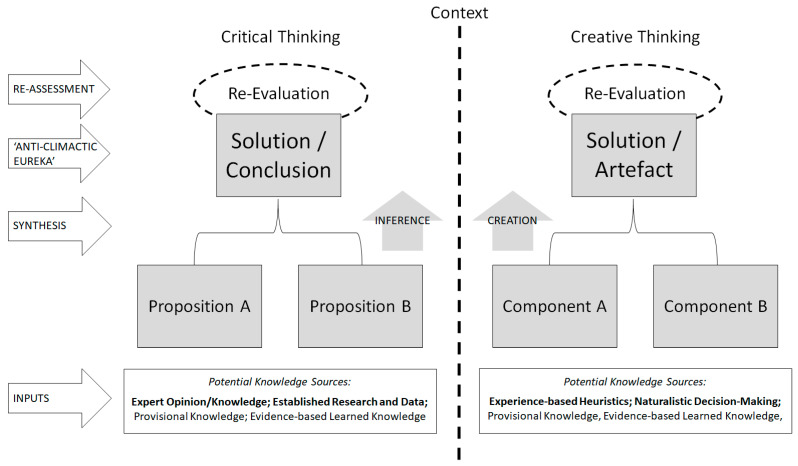
A comparative schematic of critical thinking and creative thinking in problem-solving scenarios.

Moreover, utilising CreaT in this manner may elicit some form of heightened inference ability (i.e., through the synthesis of credible, relevant and logically sound information—*connecting things*). Thus, it can be said that, in ways, certain conceptualisations of CreaT may inherently represent an established component of CritT.^4^ Figure 2 provides visual representation of the similarity between CritT and CreaT in context. Ideas are pooled and, when collated, their synthesis facilitates the generation of a solution. However, in neither case is celebration premature, despite the attractiveness of ‘eureka moments’^5^; both require further assessment, or re-evaluation. When one is concerned about the outcomes of their decision-making, regardless of whether CritT or CreaT is being engaged, caution is necessary, and such further assessment facilitates this.

### 3.2. Key Distinctions

Apart from different terminologies used between the two forms of thought, there remains much similarity between with respect to process. The only notable differences in Figure 2 are the sources from which the proposition/component pieces came and the nature of the processes’ products. Though these may seem like minor details in consideration of the structure of the processes, as presented in Figure 2, earlier discussion highlights their importance. Indeed, it is arguably the case that these two concepts are the most distinguishing when more broadly conceptualizing differences between CritT and CreaT.

With respect to input sources, to reiterate, where CritT relies on ‘more credible’ sources, based on previous evaluation, CreaT is, again, more flexible with respect to its source input. Where the two can both utilise information from credible sources, to reiterate, CreaT also encourages use of experience-based ‘evidence’, regardless of the associated emotional input that comes with it (for which it also accounts through either limiting or encouraging, depending on context; see again Section 2.2.2). Though, in this context, this is not a criticism; because the accuracy, validity, truth, quality, utility, credibility, what have you, of each component will be subject to such evaluative assessment, not only as verification (i.e., Step 7), but also at various ‘checkpoints’ throughout the series of problem-solving steps (again, see Table 1). Simply, problem-solving through CreaT does not prejudice against source-type because its focus is placed on how well the solution(s) generated overcome the identified problem, regardless of where/how the idea manifested; but like CritT, it is thorough in its evaluation.

With respect to the products of these processes, again, though they can be similar, if not the same, in many contexts; in others, they can be vastly distinct. For example, in the context of problem-solving, to reiterate, CreaT is often conceptualized as a component of CritT, in which case both processes elicit a solution (consistent with Figure 2). However, in some CritT scenarios, for example, argumentation, critically considered conclusions may be developed that utilize no creativity at all (e.g., agreeing with another’s argument without amendment, in light of thorough analysis and evaluation). Similarly, a conclusion may be drawn where the thinker abstains from drawing a conclusion (e.g., I do not know), in which case, again, nothing novel is created. On the other hand, artefacts may be developed through CreaT that extend beyond the applications of CritT and successfully satisfies its intended purpose. Simply, the products of CritT and CreaT are sometimes different.

### 3.3. Useful Complement and Further Overlap for Success

Despite such overlap presented above, it remains that there are many contexts where one of CritT or CreaT processes may be a ‘better fit’ for application than the other (e.g., when an original outcome needs to be created versus when an artefact requires analytical, evaluatory and inferential consideration); thus, such context and purpose should prioritise process use in problem-solving. However, certain contexts will also facilitate the use of both, in a complementary manner. For example, CritT might be used in a manner that identifies gaps in knowledge that might be usefully filled through CreaT. On the one hand, CreaT might yield ideas or solutions that are subsequently evaluated through CritT in a positive light and, ultimately, used. In some contexts, ideas generated creatively might *inspire* the search/identification and evaluation of a previously unconsidered evidence-base, in context. Even in scenarios where CreaT has yielded ideas that were subsequently selected against, through CritT, such ideas can facilitate useful rationales for an alternative that *is* selected.^6^. Indeed, this is an important element of CritT, where multiple perspectives are considered, in an open-minded manner, and subsequently evaluated. This is important, because from a CritT perspective, one does not simply omit evaluation of ideas because they seem poor; they are evaluated and presented, all the same, as a means of ensuring and exhibiting that various perspectives have been considered. Simply, this represents a useful strategy for communicating one’s CritT with respect to evidencing efforts made to ‘play devil’s advocate’.

Another important aspect of a ‘compromised’ complementarity for consideration is the issue of *open-mindedness* (as alluded to earlier). Halpern (2014) addresses the tendency for people to often reject new ideas (e.g., with respect to issues associated with changing one’s mind and cognitive miserliness (see Cook and Lewandowsky 2011; Kahneman 2011; Simon 1957; Tversky and Kahneman 1974)) and the propensity for creative thinkers to embrace them, in light of such ideas often being the outcomes of their CreaT. Indeed, Halpern suggests that the notion of CreaT as a cognitive process may be as simple as that of describing the process of problems-solving, the only caveat being that what is produced is novel and appropriate. Notably, if one were to accept this conceptualisation of creative thinking, then it stands to reason that CreaT should be conceptualised as an application of CritT, in as much as problem-solving.^7^ In either case, Halpern’s description alludes to a form of *open-mindedness* that is largely consistent with that pertaining to the same titled disposition towards CritT (i.e., inclinations, tendencies, or willingness to perform a given thinking skill (e.g., Quinn et al. 2020; Siegel 1999; Valenzuela et al. 2011)). In this context, disposition towards open-mindedness refers to a willingness to be cognitively flexible and avoid rigidity in thinking; tolerate divergent views and treat all viewpoints alike, prior to subsequent analysis and evaluation; detach from one’s own beliefs and consider, seriously, points of view other to one’s own without bias or self-interest; amend existing knowledge in light of new ideas/experiences; and to explore such new, alternative or unusual ideas (Dwyer et al. 2016). Interesting to note, in this context, is the reference to tasks associated with idea generation, brainstorming and engagement with new, alternative or unusual ideas—all of which have been previously described as pertinent to CreaT. Perhaps even more notable from the aforementioned research is Dwyer and colleagues’ (Dwyer et al. 2016) treatment of *disposition towards creativity*, which accepts the potential link between CritT and the tendency to visualise and generate ideas; and think differently than usual.

Furthermore, though, addressed to some extent already, perhaps the greatest barrier to understanding how CritT and CreaT overlap, differ in application, can impede one another and can co-exist in a complementary way is not engaging their conceptual descriptions beyond the ‘surface-level’. Their success, in tandem, depends on both the practical (e.g., how and when you use them), as well as the theoretical (e.g., how you conceptualise them). For example, research by Lloyd and Bahr (2010), which explored teacher perspectives on educational environments where CritT is trained, found that only 37% of academics involved in instructing or assessing CritT in university courses at least acknowledged the dispositional and self-regulatory aspects of CT; and only 47% described CT in terms of involving processes or skills. When asked about conceptualisation, one educator responded, “we expect students to do it [think critically], but now you are questioning me on my understanding of it, I wonder if I actually understand it myself”. To reiterate, likewise with respect to CreaT, conceptualisation varies from application to application; it might refer to an effortful, concerned process, as in Design and Engineering settings, or as something, ultimately, more akin to auto-heuristic idea-generation (as discussed throughout this manuscript). Thus, it may not be conceptualisation that is the issue, but rather how advocates of CritT and CreaT present and engage such conceptualisation, be it through defining terms on a theoretical level or simply showing students how it is done, on a practical level. Such consideration may play an important role with respect to generating the ‘compromise’ between them.

### 3.4. Summary and Conclusion

Overall, the perspective developed here, in light of evaluation of extant research on CritT and CreaT is that, though the two processes are often ‘lumped’ together with respect to surface-level conceptualisation, they are quite distinct. Confusing the two in application can yield some poor outcomes with respect to decision-making, idea generation and problem-solving. Simply, when applied properly, both are metacognitive, higher-order thinking processes, purposeful, goal-oriented and yield appropriate outputs, despite taking sometimes different journeys in their process and criteria for their outputs (e.g., based on originality versus evidence-based rationale).

The success of each process is dictated by context and, depending on context, one may be a ‘better fit’ for application than the other. However, this is not to say that the processes represent either–or scenarios. ‘Good thinkers’ are skilled in both—they can identify scenarios that require one or the other and appropriately utilise them. Moreover, ‘good thinkers’ know how to adapt to changing situations and recognise when to use both, in a complementary fashion (be it sequentially or synchronously), when appropriate.

Again, the efficacy of both thought forms is acknowledged, across various contexts, with potential yield of a wide-range of outcomes; thus, promotion of their use is recommended. However, caution is stressed in such promotion, as the perceived benefits, we argue, are dictated by contextual factors, such as the *how* and *when* of such application. With that, much of this consideration depends on how one conceptualises the two processes. Assuming that such conceptualisation is consistent with those provided here, then it is argued that one can, indeed, successfully infuse either process with the other, particularly with respect to ensuring the logic and appropriateness of one’s solutions.

## Figures and Tables

**Table 1:** Problem-solving checklist (adapted from Dwyer 2017; Hopwood 1974; Robbins et al. 2004).

No.	Step	Description
1	Define the problem	Analysis of the situation one believes is associated with a problem, for example, through identifying the cause(s) of the problem, desired goals and consideration of *when*, *where*, *why*, *how* and *to whom* is this problem happening.
2	Gather and organize the available information	Identification, collation and structuring of information, through analysis, as well as any other relevant knowledge (e.g., organising goals and sub-goals, obstacles, options and givens, according to the structure of the problem-solving scenario).
3	Evaluate possible strategies	Assessment of the problem space in an effort to identify a potential solution strategy (e.g., means-ends analysis, working backwards, brainstorming and constructing a problem-solving heuristic).
4	Generate possible solutions	In light of the previous steps, development of outcomes that might solve the problem; for example, through questioning their consequences, feasibility/appropriateness, and cost-effectiveness.
5	Monitor the progress of the solution strategy	Self-regulated observation, through checking and confirmation, and assessment, as needed, of the protocol’s evolution.
6	Evaluate results of the solution strategy	Re-assessment of the strategy and its outcome in light of the procedure’s output.
7	Verify the solution	Confirmation of the strategy and solutions in light of evaluation, inference of the solution, re-assessment and further amendment, if necessary.

## Data Availability

No new data were created or analyzed in this study.

## Notes

1 Further to the critical thinking skill of evaluating information through finding faults with ideas, the process can also be deemed creative in the sense that it requires imagination to develop alternatives options, possibilities and scenarios (akin to some aspects of falsification) to what has been refuted.

2 This is not a criticism by any means, but rather identification of the lasting impact of extant practice in discussing the two forms of thought together. If anything, this special issue is timely and of significant importance for creating opportunities to bring together experts in the field to formulate, compare, contrast and evaluate varying perspectives on critical and creative thinking for meaningful application.

3 Notably, CreaT perspectives presented in this manuscript are presented from the perspective of engineering, technology and design perspectives, unless otherwise stated.

4 If this is the case, one might wonder why it is not explicitly mentioned from the outset. Simply, the rationale is that doing so would reinforce the, arguably, exaggerated perceived overlap—from the outset—and, perhaps, bias readers that the distinctions that follow might be of secondary importance. As argued throughout this article, this is not the case. The distinctions are important and dictate the optimal uses of both. Moreover, such reinforcement might further disservice the conceptualisation of both processes in the sense that establishing what they are to novice audiences unfamiliar with their operational definitions is necessary to facilitate the understanding of scope and value (and not just being educational buzzwords).

5 Archimedes is often credited for coining the term *eureka*, from Ancient Greek meaning “I have found it!”. Notably, it is also the etymological root for *heuristic.* The exclamation relays excitement or relief typically associated with such intellectual discovery, breakthrough or invention. However, such romanticising of eureka moments are problematic in the sense that they imply that an idea/solution might ‘just arrive’, even though there’s no way to instantly recognise whether or not it is a good one. That is, an idea/solution must be appropriately evaluated before application and its efficacy, appropriateness or even utility verified.

6 An applied example of this, in a research setting, is the reporting of null effects. That is, an idea (hypothesis) was generated and evaluated (tested); but just because it did not work (no efficacious effect) does not mean that the null effect has no value. The reporting of the null effect is an important contribution because it provides evidence that this idea (hypothesis) was, indeed, evaluated (tested); thus, providing another link in the chain that narrows the gap between the problem-state and the goal/solution. Simply, it is a means of saying “we tried this and it did not work; so, now, let us try an alternative”.

7 Such a perspective is similar to that presented above in relation to CreaT representing a form of inference. Though problem-solving and inference refer to different concepts in CritT conceptualisation (i.e., inference is a skill and problem-solving is an application), it remains that, in both cases, it has been suggested that CreaT might represent a component of CritT.
